# Isolation and Identification of Dominant Bacteria from Raw Donkey Milk Produced in a Region of Morocco by QIIME 2 and Evaluation of Their Antibacterial Activity

**DOI:** 10.1155/2021/6664636

**Published:** 2021-08-09

**Authors:** Reda Derdak, Javier Quinteiro, Souraya Sakoui, Boutaina Addoum, Jorge Rodríguez Castro, Manuel Rey Méndez, Abdelaziz Soukri, Bouchra El Khalfi

**Affiliations:** ^1^Laboratory of Physiopathology, Molecular Genetics & Biotechnology, Ain Chock Faculty of Sciences, Health and Biotechnology Research Center, Hassan II University of Casablanca, B.P 5366 Maarif, Casablanca, Morocco; ^2^Laboratory of Molecular Systematics, Department of Biochemistry & Molecular Biology, CIBUS University of Santiago de Compostela, Santiago, Spain

## Abstract

Recently, the interest in donkey milk has increased considerably because it proved high nutritive and functional values of their ingredients. Its chemical composition is widely studied, but its microbiota, especially lactic acid bacteria, remains less studied. This study focuses on analyzing, isolating, and identifying lactic acid bacteria and evaluating their capacity to produce biomolecules with antibacterial activity. Among 44 strains identified, 43 are Gram-positive, and most are catalase-negative and cocci-shaped. Five strains were selected to evaluate their antibacterial activity against *Listeria monocytogenes, Staphylococcus aureus*, and *Escherichia coli*. Different induction methods allowed to amplify the antibacterial effects against these pathogenic strains.

## 1. Introduction

In recent decades, overuse and misuse of antibiotics has led to the emergence and spread of high bacterial resistance, making difficult to treat infections involving tremendous research efforts by the medical and scientific community [1, 2]. This concern led the international authorities to adopt concerted plans to optimize the use of antibiotics and to promote the research for alternative solutions [3]. Since, research is focused in the searching for a new bioactive molecule with antibiotic capabilities.

In fact, living organisms have developed an immense molecular diversity, containing ubiquitous low molecular weight secondary metabolites isolated from plants, insects, marine organisms, and other microorganisms [4–6], including lactic acid bacteria (LAB) [5].

LAB are microorganisms forming a group composed of bacilli and cocci [7]. The common characteristic of these bacteria is the production of lactic acid, which is the end product of the fermentation process of several sugars [8]. In addition, these germs represent a potential source of several metabolites with antimicrobial and antifungal activities like organic acids [9], reuterin, hydrogen peroxide, diacetyl [5] carbon dioxide, and bacteriocins [10–13]. They are present in different microbial biotopes such as soil, plants, the digestive system of human, and dairy products [14, 15].

Among these dairy products, we chose donkey milk (DM), which has attracted scientists' attention because of its convincing nutritive and functional elements [16, 17]. Because of its chemical composition similar to human milk [18, 19], it has therefore been used as an adequate alternative for infants with multiple food intolerances and allergies. Recently, fermented products made from DM have been proposed as important sources of probiotics and antioxidants, which have several health benefits [17, 19, 20].

Whereas chemical composition of donkey milk is very much studied, its microbiota remains less studied; therefore, this study focused (i) to analyze basic DM parameters, (ii) to isolate and identify LAB, (iii) and finally evaluate their antibacterial effect.

## 2. Materials and Methods

### 2.1. Sampling Milk

Donkey's milk sample was harvested from animals located at the Beni Mellal region, under appropriate aseptic conditions. The milk was collected in a sterile vial and transported to the laboratory at 4°C.

### 2.2. Milk Analysis

#### 2.2.1. Measurement of Milk Stability

The milk stability tests are employed to evaluate the quality of the milk and to verify the absence of chemical contamination. The tests performed are the (i) 80% v/v ethyl alcohol test, (ii) Ramsdell test, (iii) boiling test, and (iv) formalin test [21].

#### 2.2.2. Measurement of Some Physicochemical Parameters

The milk pH was determined using a pH meter [21], the acidity was determined by using a methanolic solution of phenolphthalein as a colored indicator [21], and the density was determined according to Afnor [22].

The dry matter content was estimated by evaporation in a waterbath at 70°C and then oven drying for 3 hours [21].

### 2.3. Isolation of Bacteria

The isolation was carried out by the successive depletion method on MRS medium agar and M17 medium agar (Biokar diagnostics). Seven subcultures were made until isolation of pure colonies.

#### 2.3.1. Phenotypic and Biochemical Identification

Gram stain was performed using the standard bacteriological procedure [23].

*(1) Catalase Test*. A colony was removed and then emulsified on a slide containing a drop of 3% v/v hydrogen peroxide.

*(2) Kligler Test*. Using a sterile needle, a colony was sown by simple pitting at the bottom of the agar tube (Kligler medium, Biokar diagnostics) and by tight streaks on the inclined surface of the tube, before being incubating at 32°C for 24 h. This double inoculation permits bacterial growth on the aerobic surface and in the largely anaerobic part of the tube.

*(3) Mobility Mannitol Test*. Using a sterile needle, a colony was removed and introduced into the medium up to 1 cm from the bottom of the tube, keeping the loop in the same trajectory at the entrance and at the outlet of the agar medium. Incubation was performed at 32°C for 24 h.

### 2.4. Molecular Identification

#### 2.4.1. DNA Extraction

DNA extraction was performed by a chloroform phenol method described elsewhere [24]. Quantitation of the DNA was performed using NanoDrop Lite Spectrophotometer (Thermo Fisher Scientific).

#### 2.4.2. PCR Amplification and Sequencing

Total DNA extracted from samples was used as a template for the amplification of a fragment of the 16S ribosomal DNA by polymerase chain reaction (PCR). Amplification was performed using the highly conserved universal primers: Fd1 5′-AGAGTTTGATCCTGGCTCAG-3′ and RP2 5′-ACGGCTACCTTGTTACGACTT-3′ [25].

The PCR reactions were carried out using a TC1000-G thermocycler (DLAB Scientific), in a total volume of 15 *μ*L containing approximately 100 ng of genomic DNA, 0.3 *μ*L of each 10 mm dNTP (Promega), 3 *μ*l of 5X buffer (Promega), 0.9 *μ*L of 25 mm MgCl_2_ (Promega), 0.12 *μ*L of each 10 mm primer, and 0.075 *μ*L of Taq polymerase 5 U (Promega). The PCR was programmed as follows: 95°C, 2′/(95°C, 40″-55°C, 40″-72°C, 1′) ×35; 72°C/5′/4°C.

The PCR products were purified using the ExoSAP-IT purification kit (GE Healthcare), and sequencing was performed with the BigDye Terminator Kit version 1.0 (Applied Biosystems).

Sequencing products were separated and detected in a 3730xl Genetic Analyzer (Applied Biosystems). Chromatogram revision and trimming was performed with the sequence scanner (Applied Biosystems), and alignment was obtained from ClustalX implemented in BioEdit [26].

#### 2.4.3. Taxonomic Inference

The 16SrDNA sequences were analyzed with QIIME 2 2019.10 [27]. The Sanger sequences in FASTA format were imported into a QIIME2 artefact, aligned with MAFFT [28], and a phylogenetic tree was inferred using fasttree2 [29]. The assignment to diverse taxonomic levels was performed with the q2‐feature‐classifier [30] and classify-sklearn Naïve Bayes taxonomy classifier against the Green genes 13_8 99% OTUs reference sequences [31]. In addition, each sequence was analyzed using the alignment tool BLASTN [32].

### 2.5. Antibacterial Activity

#### 2.5.1. Strains and Growth Condition

Among the strains identified, *Aerococcus viridans, Enterococcus faecalis, Enterococcus viikkiensis, Enterococcus devriesei*, and *Leuconostoc mesenteroides* were selected to evaluate their ability to produce biological active molecules. These lactic acid bacteria were cultured in MRS medium (Biokar diagnostics) and incubated 24 h at 32°C.

The pathogenic strains *Listeria monocytogenes, Staphylococcus aureus*, and *Escherichia coli* were used as indicator strains, and these strains were cultured in the Luria-Bertani (LB) medium and incubated at 37°C for 24 h.

#### 2.5.2. Optimization Assay

In order to optimize and evaluate their capacity to produce biomolecules with antimicrobial activity, the LAB were placed under different culture conditions, according to the values reported in Table 1. The incubation was performed at 32°C for 24 h.

#### 2.5.3. Diffusion of Agar

The well diffusion method was carried out following a modification of the Schillinger and Lücke [33] method. Petri dishes containing 20 mL of the LB medium supplemented with agar 0.6% (*w*/*v*) are inoculated aseptically a 10^6^ CFU/mL bacteria suspension. After solidification, 5 mm wells were created. Then, 50 *μ*L of each supernatant was deposited in each well. All dishes were put at 4°C for 4 hours and then incubated at 37°C overnight. Two negative controls were used; One containing only the indicator strain, and the other, the modified MRS medium (Table 1), to test the effect on the indicator strains. The presence of an inhibition zone (Zi) around the wells is synonymous with the production of antimicrobial substances (one or more). Zi is determined following formula:

(1) $$\begin{array}{c} \text{Zi}=\text{diameter of the Zi obtained}-\text{diameter of the well}\left(5\text{ mm}\right). \end{array}$$

## 3. Results and Discussion

### 3.1. Milk Analysis

Milk stability tests have shown that analyzed milk is stable and does not contain any chemical contamination, such as formalin and alcohol like found by other studies [34].

Physicochemical parameters are given in Table 2. The titratable acidity of the milk is 16.3°D, which explains that the milk is not altered and that the pH of the DM is neutral; this is due to the low content in casein and phosphate [35]. This result is similar to those found by Guo et al. and Salimei [36, 37]. Comparing with other milks, we find that the pH of DM is similar to human milk (7-7, 3) [19], but more basic than camel milk, cow milk, and goat milk, which is around 6 [38].

### 3.2. Microbiological Identification

The phenotypic and biochemical characteristics were evaluated for the 44 isolated bacterial strains. The morphological results showed that all these bacteria are Gram-positive, except for one strain, and with dominance for cocci shapes. Most strains are catalase negative, whereas only 17 strains showed a positive result (Table S1, supplementary material). Our results are consistent with those found by de Garnica et al. [39].

The strains were tested for their ability to use glucose and lactose in the Kligler medium [24]. All strains fermented glucose without gas production, as expected from homofermentary bacteria. None of the tested strains were able to produce H_2_S, the majority ferment lactose and mannitol, and they are immobile [24, 39].

The high level of lysozyme in DM selects for the presence of Gram-positive bacteria, as observed from the characterization of analyzed samples [40].

### 3.3. Molecular Identification

**Figure 1:**
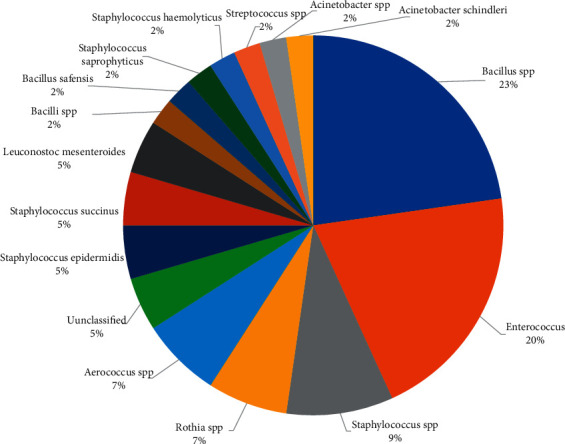
Taxonomic inference of donkey milk bacteria and the relative abundance of recovered sequences.

The molecular identification of LAB from 16S rDNA sequences showed the existence of 9 groups of bacteria (Figure 1), resolved at different taxonomic levels.

A first group is characterized by the genus *Bacillus* with 15 isolates, with a dominance of the species *Bacillus amyloliquefaciens*, as inferred from the BLASTN search. A second group was characterized by the genus *Enterococcus* with 9 isolates. A third group included 7 isolates from the genus *Staphylococcus*. Three isolated from the identified *Aerococcus viridans* were included in a fourth group. A fifth group was characterized by the genus *Leuconostoc* with a frequency of 2 isolates. The other groups included a single isolate, *Rothia*, *Acinetobacter*, and *Streptococcus*.

The taxonomic inference combining a BLASTN search and the importation of moderate Sanger sequence data into the standard NGS metagenomic pipeline resulted in congruent and reliable taxa assignation.

All these species have been previously isolated and studied from dairy products, such as cow's milk, goat's milk, and sheep's milk [39, 41–45]. Another study aimed at characterizing the bacterial community of donkey milk and showed that the basic microbiota was composed of genera including LAB and species typically present in plants, soil, and water [46].

### 3.4. Antibacterial Activity

The LABs were put under different culture conditions to optimize the production of the antibacterial substances; the inhibitory spectra of these bacteria are given in Table 3.

These results reveal that the supernatants of the 5 studied strains show an inhibition against the pathogenic indicator strains, with the exception of *Aerococcus viridans* and *Enterococcus viikkiensis.* These species showed a weak inhibition under different culture conditions against *Staphylococcus aureus* and *Escherichia coli*, respectively. All strains exhibited significant inhibition against *Listeria monocytogenes*. The maximum activity was observed with Tween 20, UV, and in coculture with *Listeria* with diameters of 28, 20, and 21 mm respectively. Subsequent studies have demonstrated the ability of Tween 20 to increase bacteriocin production and as a result demonstrated inhibitory activity in its presence [47–49]. Furthermore, another study has shown that the regulation of bacteriocin expression depends on an external stimulus such as cocultivation with other strains [50]. Referring to the controls (Table S2), the inhibitory activity observed in the presence of SDS appears to be caused by SDS itself and not by lactic acid strains. This hypothesis has been confirmed by Chen and Yanagida [51].

## 4. Conclusion

The present study has shown that the five lactic strains tested are capable of producing active biomolecules with inhibitory activity on the pathogenic strains: *Listeria monocytogenes*, *Escherichia coli*, and *Staphylococcus aureus*. This antibacterial activity could be due to the production of bacteriocins or other biomolecules. Studies are underway to characterize and identify these antimicrobial compounds.

## Figures and Tables

**Table 1:** The different culture conditions for the evaluated strains.

Methods	Modified media
1	2.5% yeast extract
2	0.5% glucose
3	Manganese (0.033 mm)
4	Ultraviolet
5	SDS
6	1% Tween 20
7	Coculture with *L. monocytogenes*
8	EDTA

**Table 2:** Results of physicochemical parameters of donkey's milk.

Test	Result
pH	7.1
Titrable acidity	16.3°D
Dry matter	127 g/L
Density	1,032

**Table 3:** Antibacterial activity of supernatant from lactic acid bacteria against pathogenic bacteria.

Strains		Culture condition								
		MRS	MRS + E.Y.	MRS + glu	MRS + Mn	UV	MRS + SDS	MRS + Tw20	MRS + Listeria	MRS + EDTA
*Aerococcus viridans*	*Escherichia coli*	+	+	+	+++	+	+++	++++	+++	+
	*Staphylococcus aureus*	+	+	+	+	+	+	++	+	+
	*Listeria monocytogenes*	++	++	+++	+	++++	+++	++++	++++	+
*Enterococcus faecalis*	*Escherichia coli*	+	+	+	+	++	+	+++	++	+
	*Staphylococcus aureus*	++	+	+	++	++	++	++	+	++
	*Listeria monocytogenes*	++	+++	+++	+	+++	++	++++	++	+
*Enterococcus devriesei*	*Escherichia coli*	+	+	+	+	++	+	+	++++	+++
	*Staphylococcus aureus*	+	++	++	++	++	++	++	++	+
	*Listeria monocytogenes*	+	+	++	++	+++	++	++++	+++	+
*Enterococcus viikkiensis*	*Escherichia coli*	+	+	+	+	+	+	+	+	+
	*Staphylococcus aureus*	+	+	++	++	++	+	++	++++	+
	*Listeria monocytogenes*	++	+++	++++	+++	++++	+	++++	++++	+
*Leuconostoc mesenteroides*	*Escherichia coli*	++	++	+++	++++	++++	+	+	+	+
	*Staphylococcus aureus*	++	++	++	+	++	+	++	++	+
	*Listeria monocytogenes*	+	++++	++	++	++	+	++++	++++	+

E.Y., extract yeast; Glu, glucose; Mn, manganese; UV, ultraviolet; SDS, sodium dodecyl sulfate; Tw20, Tween 20. Antibacterial activity was expressed as growth inhibition, low (+), moderate (++), or strong (+++). (+) weak inhibition (4–9 mm), (++) intermediate inhibition (10–14 mm), (+++) strong inhibition (14–19 mm), and (++++) very strong inhibition (>19 mm).

## Data Availability

The microbiological and molecular data used to support the findings of this study are available from the corresponding author upon request.

## Supplementary Materials

Supplementary Materials The supplementary material of the research article “Isolation and identification of bacterial population of donkey milk from a region of Morocco by QIIME 2 and evaluation of their biomolecules” by Derdak et al. is composed by two tables. Table S1: phenotypic and biochemical characteristics of isolated lactic bacteria. Table S2: blanks for *Staphylococcus aureus*.
