# Supplementary material for: Isolation and Identification of Dominant Bacteria from Raw Donkey Milk Produced in a Region of Morocco by QIIME 2 and Evaluation of Their Antibacterial Activity
Source: ScientificWorldJournal. 2021 Aug 9;2021:6664636. doi: 10.1155/2021/6664636 (PMC8371658; doi:10.1155/2021/6664636)
Supplement: Supplementary Materials — The supplementary material of the research article “Isolation and identification of bacterial population of donkey milk from a region of Morocco by QIIME 2 and evaluation of their biomolecules” by Derdak et al. is composed by two tables. Table S1: phenotypic and biochemical characteristics of isolated lactic bacteria. Table S2: blanks for Staphylococcus aureus. [file 6664636.f1.docx]

Supplementary files

Table S1 : Phenotypic and Biochemical Characteristics of Isolated Lactic Bacteria

H2S: Hydrogen sulfide, +: Positive test, -: Negative test.

|  | Gram | Shape | Catalase | Glucose | Lactose | mannitol | Mobility | CO_2_ | H_2_S |
| --- | --- | --- | --- | --- | --- | --- | --- | --- | --- |
| L1 | + | Cocci | - | + | + | + | - | - | - |
| L2 | + | Cocci | - | + | + | + | - | - | - |
| L3 | + | Cocci | - | + | + | + | - | - | - |
| L4 | + | Cocci | - | + | + | + | - | - | - |
| L5 | + | Bacilli | - | + | - | - | - | - | - |
| L6 | + | Cocci | - | + | + | + | - | - | - |
| L7 | + | Cocci | - | + | + | + | - | - | - |
| L8 | + | Cocci | - | + | + | + | - | - | - |
| L9 | + | Cocci | - | + | + | + | - | - | - |
| L10 | + | Cocci | - | + | + | + | - | - | - |
| L11 | + | Cocci | - | + | + | + | - | - | - |
| L12 | + | Cocci | + | + | + | + | - | - | - |
| L13 | + | Cocci | + | + | + | + | - | - | - |
| L14 | + | Cocci | - | + | + | + | - | - | - |
| L15 | + | Cocci | - | + | + | + | - | - | - |
| L16 | + | Cocci | + | + | + | + | - | - | - |
| L17 | + | Cocci | + | + | + | + | - | - | - |
| L18 | + | Bacilli | - | + | - | - | - | - | - |
| L19 | + | Cocci | - | + | + | + | - | - | - |
| L20 | + | Cocci | - | + | + | + | - | - | - |
| L21 | + | Cocci | - | + | + | + | - | - | - |
| L22 | + | Cocci | - | + | + | - | - | - | - |
| L23 | + | Bacilli | - | + | + | + | + | - | - |
| L24 | + | Bacilli | - | + | + | + | + | - | - |
| L25 | + | Cocci | - | + | + | - | - | - | - |
| L26 | + | Cocci | - | + | + | + | - | - | - |
| L27 | + | Cocci | - | + | + | + | - | - | - |
| L28 | + | Cocci | - | + | + | + | - | - | - |
| L29 | + | Bacilli | - | + | + | + | + | - | - |
| L30 | + | Cocci | - | + | + | - | - | - | - |
| L31 | + | Cocci | + | + | + | - | - | - | - |
| L32 | + | Cocci | - | + | + | + | - | - | - |
| L33 | + | Cocci | + | + | + | + | - | - | - |
| L34 | + | Bacilli | + | + | + | + | + | - | - |
| L35 | + | Bacilli | + | + | + | + | + | - | - |
| L36 | + | Bacilli | + | + | + | - | + | - | - |
| L37 | + | Bacilli | + | + | + | - | + | - | - |
| L38 | + | Bacilli | + | + | + | - | + | - | - |
| L39 | + | Bacilli | + | + | + | - | + | - | - |
| L40 | + | Bacilli | + | + | + | - | + | - | - |
| L41 | + | Bacilli | + | + | + | - | + | - | - |
| L42 | + | Bacilli | + | + | + | - | + | - | - |
| L43 | - | Cocci | + | + | + | - | + | - | - |
| L44 | + | Bacilli | - | + | + | + | + | - | - |

| Conditions | MRS | MRS+Tween 20 | MRS +SDS | MRS + EDTA |
| --- | --- | --- | --- | --- |
| Zone of inhibition | 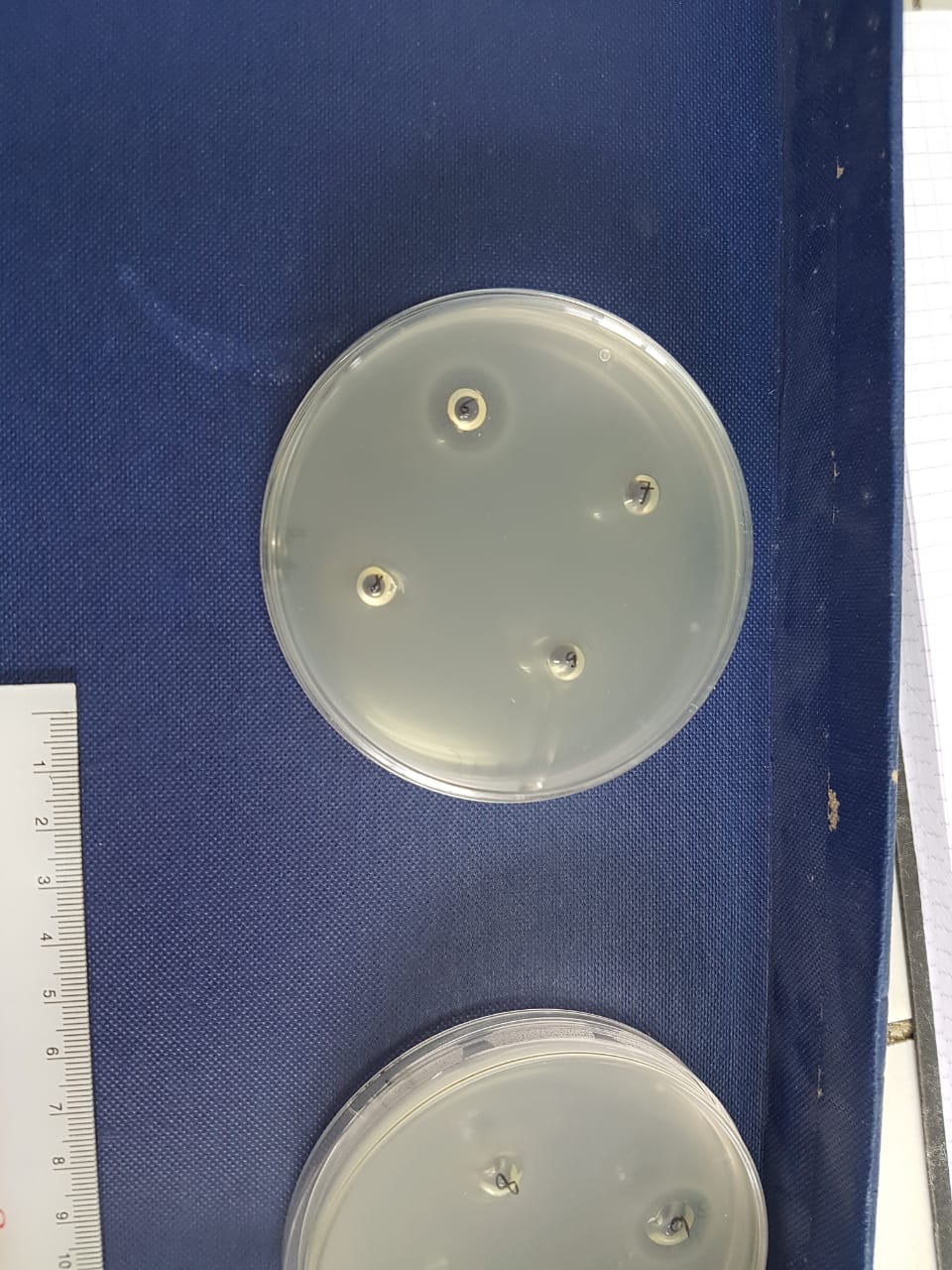 | 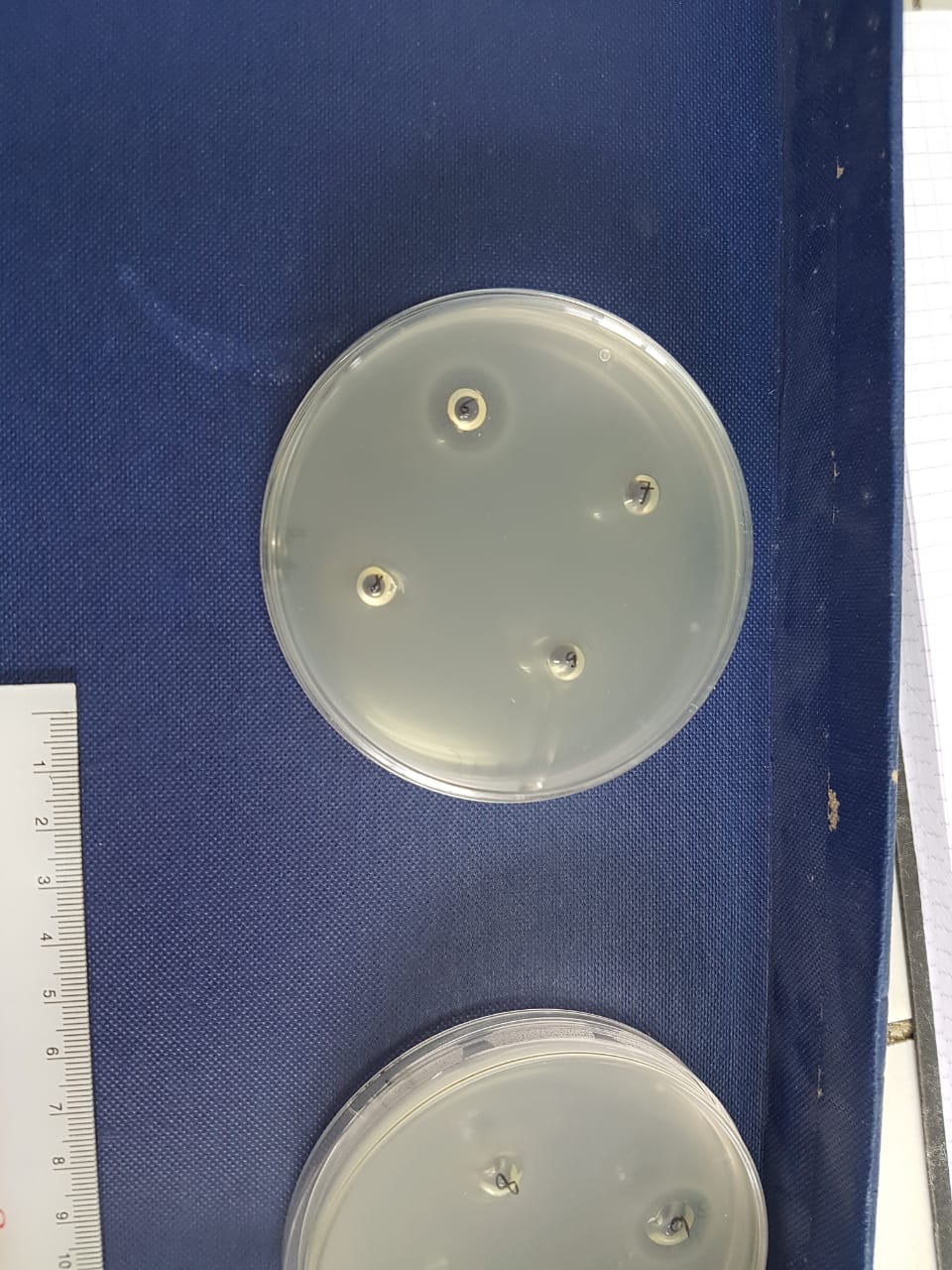 | 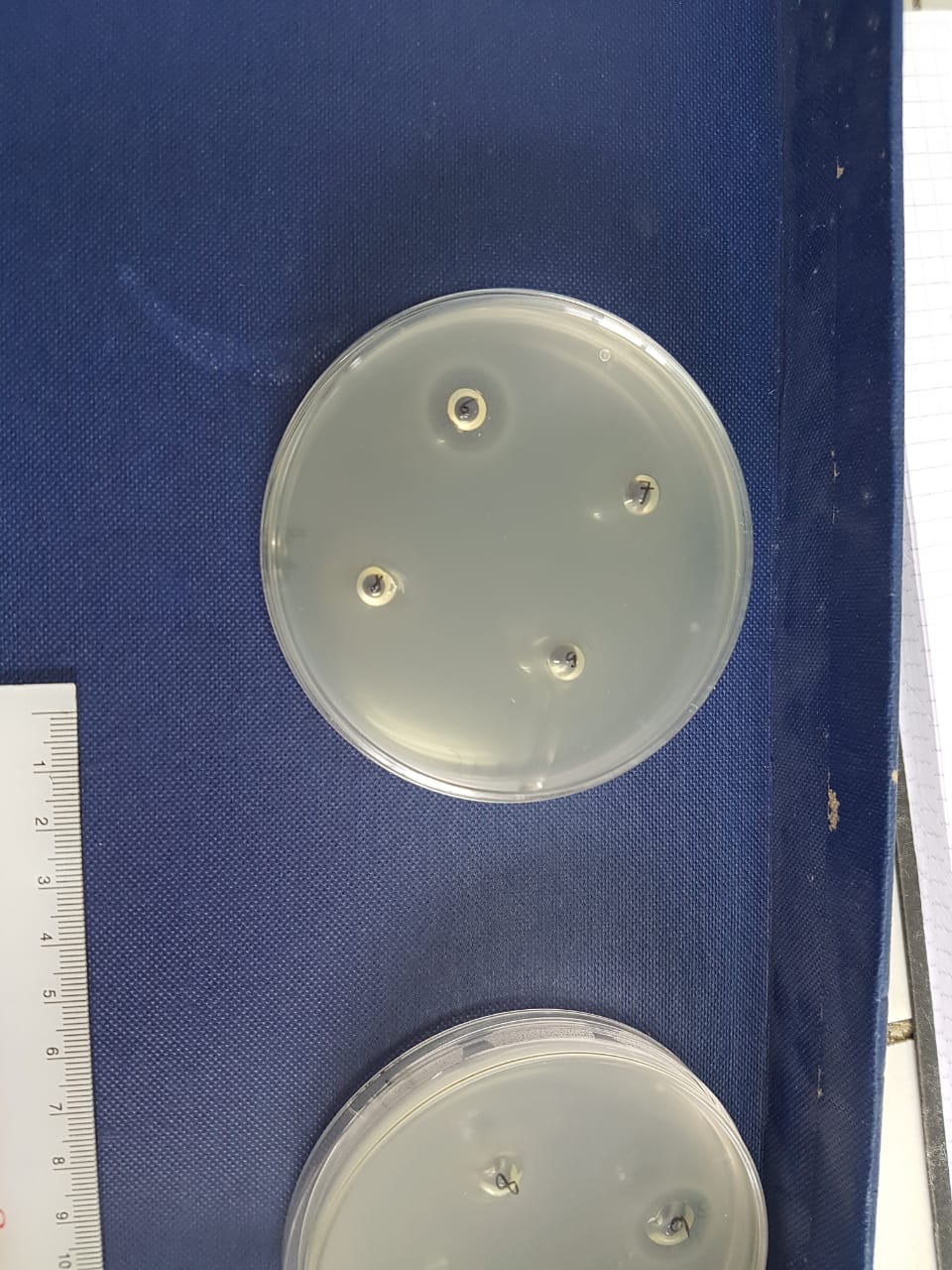 | 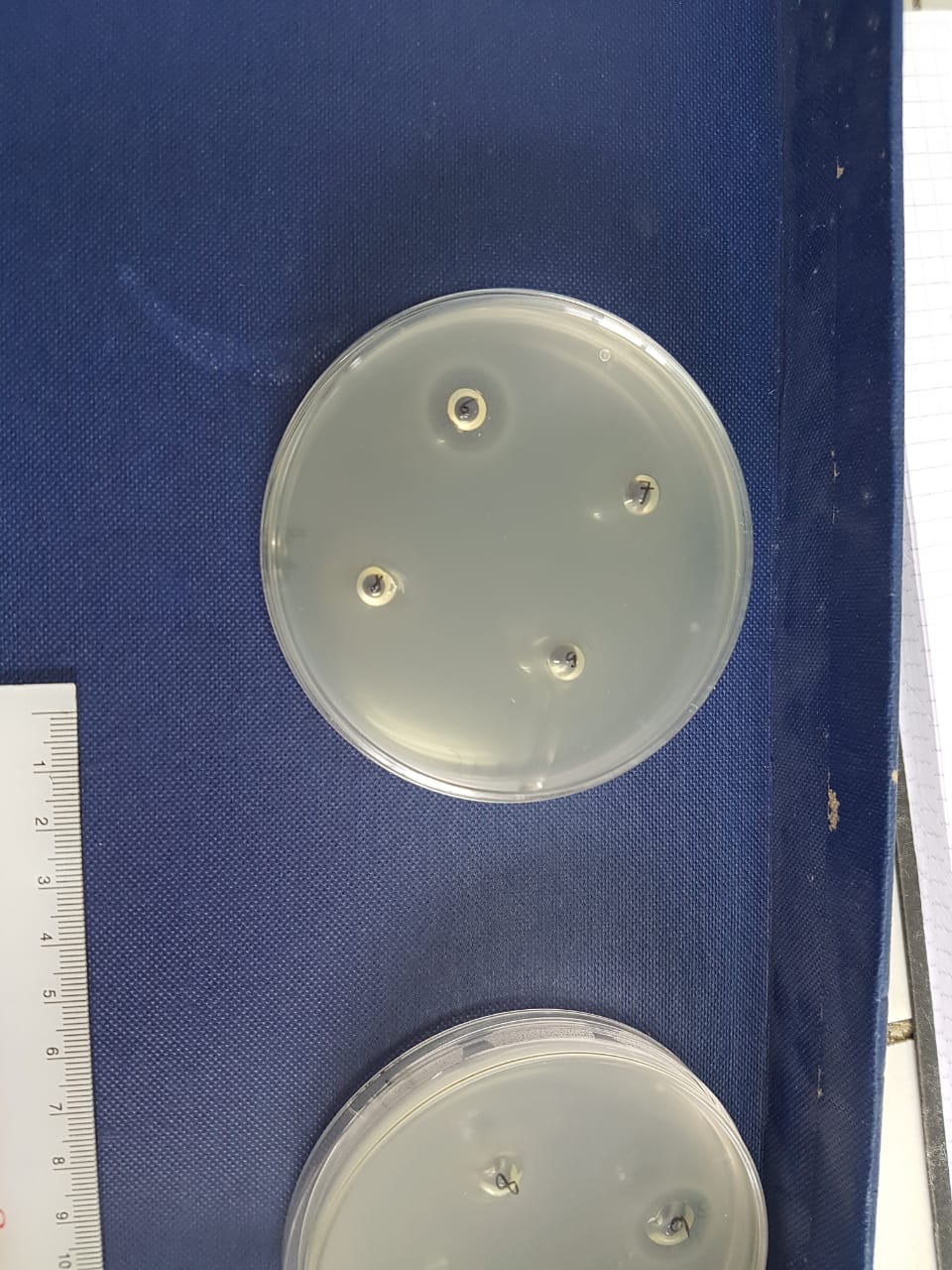 |
| Diameter ( mm) | 0 | 0 | 11 | 0 |

Table S2: blancs for staphylococcus aureus
